# Facial tremor in patients with Parkinson’s disease: prevalence, determinants and impacts on disease progression

**DOI:** 10.1186/s12883-021-02105-y

**Published:** 2021-02-23

**Authors:** Ruwei Ou, Qianqian Wei, Yanbing Hou, Lingyu Zhang, Kuncheng Liu, Junyu Lin, Zheng Jiang, Bi Zhao, Bei Cao, Huifang Shang

**Affiliations:** Department of Neurology, Laboratory of Neurodegenerative Disorders, West China Hospital, Sichuan University, Chengdu, 610041 Sichuan China

**Keywords:** Parkinson’s disease, Facial tremor, Rest tremor, Prevalence, Prognosis

## Abstract

**Background:**

Facial (lip and jaw) tremor (FT) is associated with Parkinson’s disease (PD) but few studies have been conducted to explore its clinical profile. We performed this study to investigate the prevalence and clinical correlates of FT in PD, and further to evaluate its effect on disease progression.

**Methods:**

A retrospective, cross-sectional (*n* = 2224) and longitudinal (*n* = 674) study was conducted. The presence of FT was based on a ≥ 1 score in the United PD Rating Scale (UPDRS) item 20A. Group comparisons were conducted, followed by a forward binary logistic regression analysis. Inverse probability of treatment weighting (IPTW) based on the propensity score and weighted or unweighted Cox regression models were used to explore the impact of FT on five clinical milestones including death, UPDRS III 11-point increase, Hoehn and Yahr (H&Y) stage reaching 3, dyskinesia development, and Montreal Cognitive Assessment 3-point decrease.

**Results:**

FT was presented in 403 patients (18.1%), which showed increasing trends with disease duration and H&Y score. Age (*P* < 0.001), female (*P* < 0.001), disease duration (*P* = 0.001), speech (*P* = 0.011), rigidity (*P* = 0.026), rest tremor on limbs (*P* < 0.001), kinetic tremor on hands (*P* < 0.001), and axial symptoms (*P* = 0.013) were independent factors associated with FT. Both unweighted and weighted Cox regression models indicated that baseline FT and FT as the initial symptom were not associated with the five outcomes.

**Conclusions:**

Our study suggested that FT was not uncommon and provided a deeper insight into the characteristics of FT in PD. The predict value of FT on long-term progronis of PD may need future longer follwe-up study.

**Supplementary Information:**

The online version contains supplementary material available at 10.1186/s12883-021-02105-y.

## Background

Parkinson’s disease (PD) is a chronic, progressive neurodegenerative disease mainly characterized by rest tremor, rigidity, bradykinesia, and postural and gait disorders [1]. As one of the core motor symptoms, rest tremor is reported in most of the cases (74.4%) at disease onset [2] and occurs more prevalently in the upper limb rather than lower limb and face [3]. Facial (lip and jaw) tremor (FT) at rest is considered as a relatively uncommon symptom of PD, which can be seen in the early stage of the disease [4]. It is reported that only 1.7% of patients had FT at disease onset, and its prevalence could be up to 14% after a mean 9-year disease duration [5]. FT can cause knocking of the teeth, resulting in a vexing sound and social embarrassment, and contribute to the reduced quality of life of patients [6].

The exact functional anatomy of rest tremor in PD remains unclear. It is probably related to a combined impairment of the cerebello-dentato-thalamocortical and the basal ganglia–thalamocortical circuits [7]. Rest tremor is often less responsive to dopamine replacement therapy than bradykinesia and rigidity, and its responsiveness to dopaminergic treatment is quite variable among PD patients [8]. In some cases, FT in PD is thought to not respond well to dopaminergic drugs [6]. However, there is also evidence that FT can respond to dopaminergic therapy and has predictive value for clinical PD diagnosis [4]. These controversial results suggest that dopamine deficiency alone may do not determine FT severity, and its response to dopaminergic therapy is likely influenced by multiple factors.

To date, data on the relationships between FT with other PD motor and non-motor symptoms are scarce, and the prevalence of FT is based on a few studies with a small sample size. Although previous analyses of some studies reported that patients with tremor-predominant (TD) subtype have a more benign course [9] and show less severe cognitive deficits [10], a recent study verified that there was no evidence of a benign effect of tremor [11]. So far, it is still unsure the association between FT and PD progression. Thus, we aimed to explore the prevalence and clinical correlates of FT in a large cohort of Chinese PD patients, and then followed up a group of patients at an early stage to further examine whether FT can act as a useful predictor for the progression of PD.

## Methods

### Participants

All procedures of the current study were approved by the Ethics Committee of West China Hospital, Sichuan University. Initially, we enrolled 4332 PD patients from the Department of Neurology, West China Hospital of Sichuan University between March 2009 and July 2019. All participants have provided written informed consent and met the Unified Kingdom PD Society Brain Bank Clinical Diagnostic Criteria for PD [12]. To investigate the prevalence and clinical correlates of FT, we limited our study sample in patients who were assessed at “off” medication (*n* = 2224).

To explore the influence of FT on the disease progression, participants who met the following inclusion criteria were followed up at least once (range 1–10) (*n* = 725): 1) assessment at “off” medication; 2) disease duration < 3 years; 3) Hoehn and Yahr (H&Y) stage < 3; 4) absence of motor complications; and 5) absence of dementia. During the follow-up visit, 21 patients withdraw informed consent and 30 patients lost contact, resulting in the remaining 674 patients who provided information on clinical outcomes were included in the longitudinal data analysis.

### Clinical assessments

Trained neurologists completed standardized assessments of all patients. Demographic and clinical data including sex, age, age at onset, disease duration, education, smoking and drinking history, hypertension, and diabetes mellitus (DM) history, motor complications, and total levodopa equivalent daily dosage (LEDD) were collected. The LEDD was calculated based on a previous systematic review [13]. The Unified PD Rating Scale (UPDRS) part III [14] and H&Y stage (range 1–5) [15] was used to evaluate the motor symptoms, which was divided into speech, facial expression, tremor at rest, action or postural tremor of hands, rigidity, bradykinesia, and axial symptoms. If possible, all patients were indicated to withdraw medications > 12 h at follow-up visit (*n* = 489, 72.6%). For those without an “off” score, we estimated an “off” medication score by adding the difference value of the study population’s mean “off”- and mean “on”-scores to the patient’s “on” medication score, as reported by a previous study [16].

The global non-motor symptoms (NMS) was assessed using the Chinese version of the Non-Motor Symptoms Scale (NMSS) [17]. In addition, cognitive function was measured using the Frontal Assessment Battery (FAB; range 1–18) [18] and Montreal Cognitive Assessment (MoCA; range 0–30) [19], with lower scores indicating poor cognition. Depression and anxiety were assessed using the Hamilton Depression Rating Scale (HDRS) (24 items) [20] and the Hamilton Anxiety Rating Scale (HARS), respectively [21]. Freezing of gait was assessed based on scored ≥1 on the Freezing of gait Questionnaire item-13 “Do you feel that your feet get glued to the floor while walking, making a turn, or when trying to initiate walking?” [22]. Falls were determined either with a ≥ 1 score on UPDRS-13 (falling unrelated to freezing) or a ≥ 3 score on UPDRS-14 (falling related to freezing).

### Determination of FT

The diagnosis of FT was established by a neurologist trained in movement disorders, and presence or absence explicitly marked on the UPDRS-III score sheet (item 20A: tremor at rest: face, lips, and chin).

### Clinical outcomes

#### Survival

Mortality surveillance was performed mainly throughout the continuing active follow-up of patients and their families. It lasted until July 1, 2020, which was 11 years after our study began (2009), with as many as 14 years of passive follow-up for mortality for patients first diagnosed in 2006.

#### Motor decline

Since a change of 2.5–5.2 points on the UPDRS III score represent a clinically significant difference [23], we thus defined a fast motor progression, based on the mean 3.7 ± 2.2 years of follow-up, as an 11-point increased in the UPDRS-III score (mean 3-point per year) and time to such event as the time from the baseline to follow-up visit in which an 11-point increase was first measured.

#### H&Y stage

Time to transfer to H&Y stage 3 was defined as the time from the baseline to the first follow-up examinations in which the patient scored at least stage 3.

#### Dyskinesia

Time to dyskinesia development was defined as the time from the baseline to the first follow-up examinations in which the patient reported dyskinesia.

#### Cognitive decline

Cognitive decline was defined as a 3-point decrease from baseline MoCA score and time to event as the time from the baseline to follow-up examinations in which a 3-point decrease was first measured.

### Statistical analyses

Continuous variables were presented as mean ± standard deviation if normally distributed and as median (interquartile range) if non-normally distributed. Categorical data were shown as number (percentage). To identify the differences in demographic and clinical features at baseline between patients with and without FT, the Student’s *T* test, Wilcoxon rank-sum test, or chi-square test were applied as appropriate.

To explore the potential clinical factors related to FT, a binary logistic regression model was used. The presence or absence of FT was set as the dependent variable and the variables that reported differences between the two groups (selection criterion *P* < 0.1) or those that were possibly related to FT were chosen as independent variables. The Hosmer and Lemeshow test was implemented to examine the goodness of fit, with *P* value > 0.05 suggesting high goodness of fit of the model. The tolerance and variance inflation factor (VIF) that were calculated based on a multiple linear regression analysis were used to diagnose the multicollinearity among each independent variable, with tolerance < 0.2 or VIF > 5 suggesting the presence of multicollinearity.

Because of significant differences in various baseline characteristics between patients with and without FT (or patients with and without an initial symptom of FT), a propensity score (PS) weighting method was served to balance the differences. The PS model was developed by constructing a logistic regression model in which patients with FT vs. patients without FT (or patients with and without an initial symptom of FT) were regressed on baseline characteristics related to the outcome variables. The estimated PS was achieved as the predicted probability of having FT in each subject. The inverse probability of treatment weighting (IPTW) [24] was then calculated as the inverse of the PS for the patients with FT and as the inverse of (1 - PS) for the patients without FT. To assess bias reduction achieved by the PS weighting, standardized mean differences (SMD) of the confounding covariates that were included for estimating PS were compared between patients with and without FT before and after weighting, with a value of < 10% indicating between-group balance. All subsequent analyses were weighted by IPTW.

Furthermore, to verify the results from IPTW, a sensitivity analysis was conducted. Multivariate Cox proportional hazards regression models were conducted to calculate hazard ratios (HRs) and 95% confidence intervals (CIs) of five clinical outcomes across either presence or absence of FT or FT as the initial symptom or not. Two models were conducted. In model 1, sex, age, and age at onset were adjusted. In model 2, sex, age, age at onset, BMI, LEDD, UPDRS III score, MoCA score, and NMSS score were adjusted.

Statistical analyses were conducted by R version 4.0.0 using “Matching”, “survey”, “reshape2”, “survival”, and “reportReg” packages. All statistical tests were two-tailed, and *P* values < 0.05 were regarded as statistically significant.

## Results

### Prevalence of FT

**Fig. 1:**
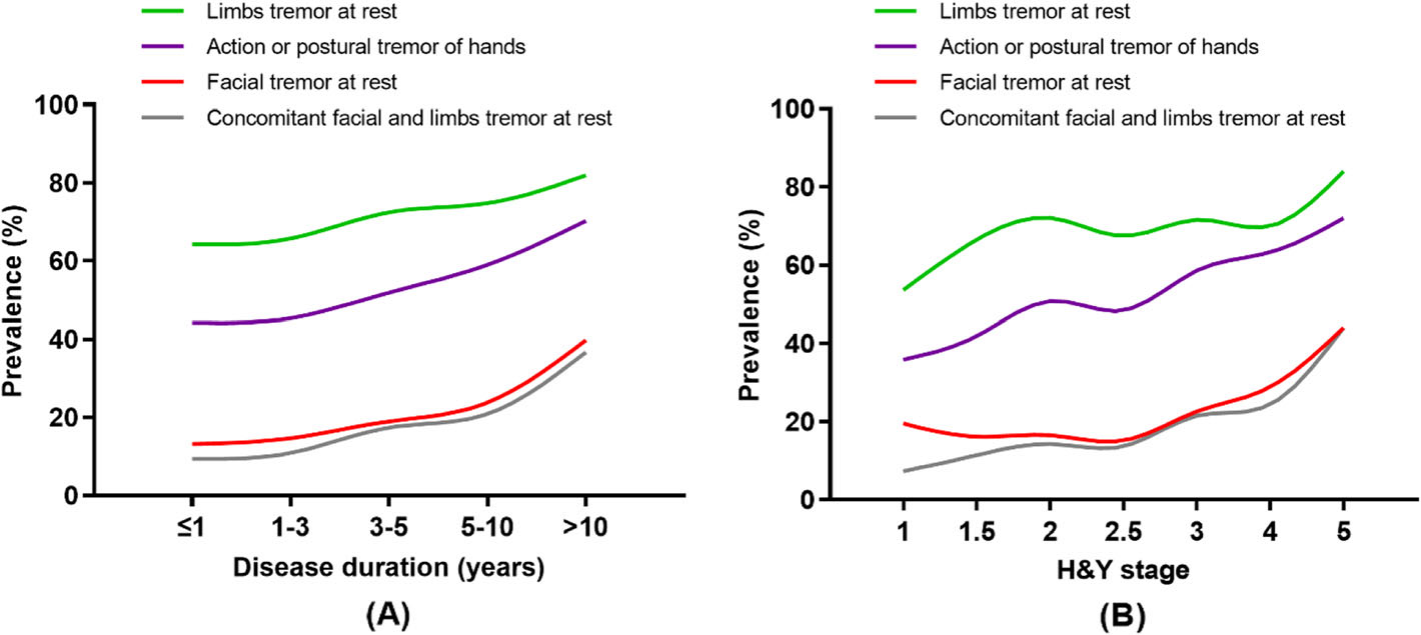
Prevalence of tremor in patients with PD with different disease duration and H&Y stage. The prevalence of each phenotype of tremor including facial tremor at rest, concomitant facial and limbs tremor at rest, limbs tremor at rest as well as action or postural tremor of hands showed an increased tendency with the increased disease duration (**a**) and H&Y stage (**b**)

In total, 2224 PD patients (1181 males and 1043 females) were included for retrospective analysis. Among these patients who were assessed UPDRS at “off” medication, 403 patients reported FT (18.1%). The smooth curves indicated that the prevalence of FT along with limbs tremor at rest or action/postural tremor of hands showed an increasing tendency with increased disease duration and H&Y stage (Fig. 1).

### Comparison between patients with and without FT

The mean age of the included patients at enrollment was 62.3 ± 9.9 years, with a mean onset age of 58.9 ± 9.9 years and a mean disease duration of 3.4 ± 4.3 years (Table 1). The mean UPDRS-III score after exclusion of the FT score was 29.2 ± 14.2. Compared with patients without FT, those with FT had significantly lower education, older age, older age of onset, longer disease duration, higher UPDRS III score (exclusion of FT score) as well as higher sub-scores of speech, rest tremor at hands, action or postural tremor, bradykinesia, and axial symptoms, greater H&Y stage, and lower FAB and MoCA score (*P* < 0.05) (Table 1).

**Table 1:** Demographic and clinical features between PD patients with and without FT

	Total (*n* = 2224)	With FT (*n* = 403)	Without FT (*n* = 1821)	*P*-value
Education	9.5 ± 4.1	9.0 ± 4.3	9.6 ± 4.0	0.016*
Sex, male	1181 (53.1%)	171 (42.4%)	1010 (55.5%)	< 0.001*
Age	62.3 ± 9.9	65.4 ± 9.7	61.6 ± 9.9	< 0.001*
Age at onset	58.9 ± 9.9	60.8 ± 10.1	58.5 ± 9.9	< 0.001*
Disease duration	3.4 ± 3.4	4.5 ± 4.2	3.1 ± 3.1	< 0.001*
LEDD	232.0 ± 275.7	241.6 ± 310.0	229.9 ± 267.6	0.485
UPDRS-III^a^	29.2 ± 14.2	32.5 ± 17.4	28.4 ± 13.3	< 0.001*
Speech	0.7 ± 0.7	0.8 ± 0.8	0.7 ± 0.7	< 0.001*
Facial expression	1.5 ± 0.8	1.5 ± 0.9	1.5 ± 0.8	0.215
Tremor at rest^a^	2.2 ± 2.4	3.3 ± 2.9	1.9 ± 2.1	< 0.001*
Action or postural tremor	1.6 ± 1.4	2.2 ± 1.9	1.4 ± 1.3	< 0.001*
Rigidity	8.0 ± 3.9	8.1 ± 4.4	8.0 ± 3.8	0.607
Bradykinesia	12.3 ± 6.8	13.2 ± 7.7	12.0 ± 6.6	< 0.001*
Axial symptoms	3.0 ± 2.7	3.3 ± 3.1	2.9 ± 2.5	0.006*
H&Y stage	2.0 (0.5)	2.0 (0.5)	2.0 (0.5)	0.025*
Freezing of gait	462 (20.8%)	95 (23.6%)	367 (20.2%)	0.126
Falls	151 (6.8%)	32 (7.9%)	119 (6.5%)	0.310
Motor fluctuation	212 (9.5%)	88 (14.7%)	340 (12.2%)	0.087
Dyskinesia	76 (3.4%)	33 (5.5%)	169 (6.0%)	0.623
FAB	15.2 ± 2.7	14.8 ± 2.8	15.3 ± 2.7	0.001*
MoCA	22.9 ± 4.8	22.0 ± 5.1	23.1 ± 4.7	< 0.001*
HDRS	9.4 ± 8.2	9.8 ± 8.1	9.3 ± 8.2	0.244
Depression	727 (32.7%)	145 (36.0%)	582 (32.0%)	0.120
HARS	7.2 ± 6.6	7.3 ± 6.7	7.1 ± 6.6	0.631
Anxiety	653 (29.4%)	128 (33.8%)	525 (30.8%)	0.242
NMSS	35.7 ± 30.5	39.0 ± 32.9	34.9 ± 29.9	0.024*

*PD* Parkinson’s disease, *FT* Facial tremor, *LEDD* Levodopa Equivalent Daily Doses, *UPDRS* Unified PD Rating Scale, *H&Y stage* Hoehn and Yahr stage, *FAB* Frontal Assessment Battery, *MoCA* Montreal Cognitive Assessment, *HDRS* Hamilton Depression Rating Scale, *HARS* Hamilton Anxiety Rating Scale, *NMSS* Non-Motor Symptoms Scale

^a^The total score was calculated after excluding the score of FT at rest

* Significant difference

### Correlative factors of FT

**Fig. 2:**
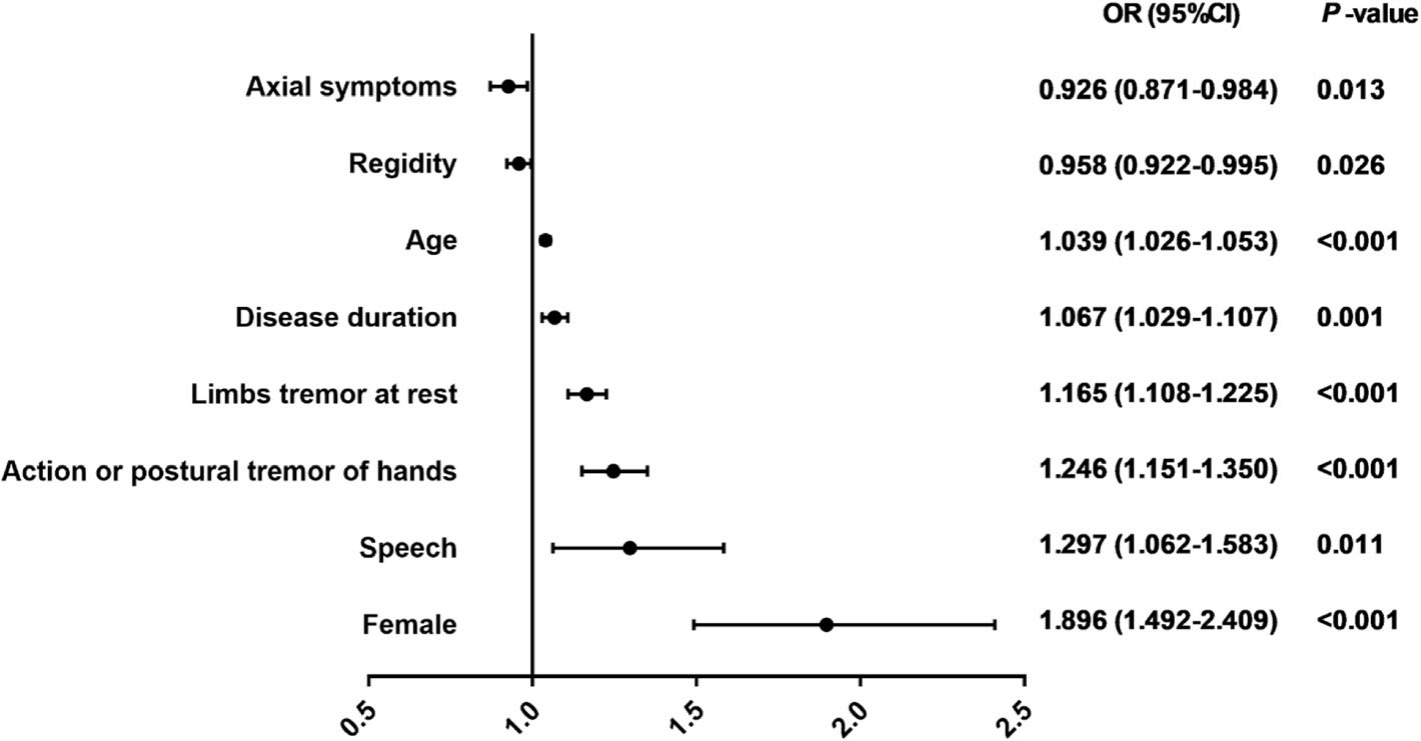
Correlative factors of facial tremor at rest in patients with PD. Forest map showed correlative factors of facial tremor at rest in a group of PD patients who were assessed at “off” medication state. In this model, the following controlled covariates were non-significant: education, facial expression score, bradykinesia score, FAB score, MoCA score, and NMSS score. All OR, 95%CI, and *P*-values were calculated from a forward binary logistic regression analysis

Forest map indicated that FT was association with age (OR = 1.039, 95%CI = 1.026–1.053, *P* < 0.001), females (OR = 1.896, 95%CI = 1.492–2.409, *P* < 0.001), disease duration (OR = 1.067, 95%CI = 1.029–1.107, *P* = 0.001), speech (OR = 1.297, 95%CI = 1.062–1.583, *P* = 0.011), rigidity (OR = 0.958, 95%CI = 0.922–0.995, *P* = 0.026), limbs tremor at rest (OR = 1.165, 95%CI = 1.108–1.225, *P* < 0.001), action or postural tremor of hands (OR = 1.246, 95%CI = 1.151–1.350, *P* < 0.001), and axial symptoms (OR = 0.926, 95%CI = 0871–0.984, *P* = 0.013) (Fig. 2). The tolerance of each independent variable was more than 0.2 and all the VIF were less than 5 (Supplementary Table 1), suggesting there was no multicollinearity presented in the model. The Hosmer and Lemeshow test showed that the goodness of fit of the model was superior (*P* = 0.686). The percentage accuracy in the classification of the model was 81.9%.

### Effects of FT on clinical outcomes

Of the 674 participants who completed the follow-up visits, 65 patients died (9.6%), with a mean time to death or censoring from baseline of 5.7 ± 2.3 years; 203 (30.1%) reported a decrease of at least 11 points in UPDRS III score after 3.7 ± 2.2 years of follow up; 119 (17.7%) patients reached at least H&Y stage 3 after a mean disease duration of 4.9 ± 2.0 years; 161 (23.9%) patients reported a decrease of at least 3 points in MoCA score after a mean 3.6 ± 2.1 years of follow-up; and 94 (13.9%) reported new occurrence of dyskinesia after a mean 5.1 ± 2.1 years of follow up. The mean change in the UPDRS III score from baseline to follow-up visit was − 7.9 ± 10.8 points, while the mean change in the MoCA score was − 0.7 ± 3.1 points, after excluding the patients who had died.

**Fig. 3:**
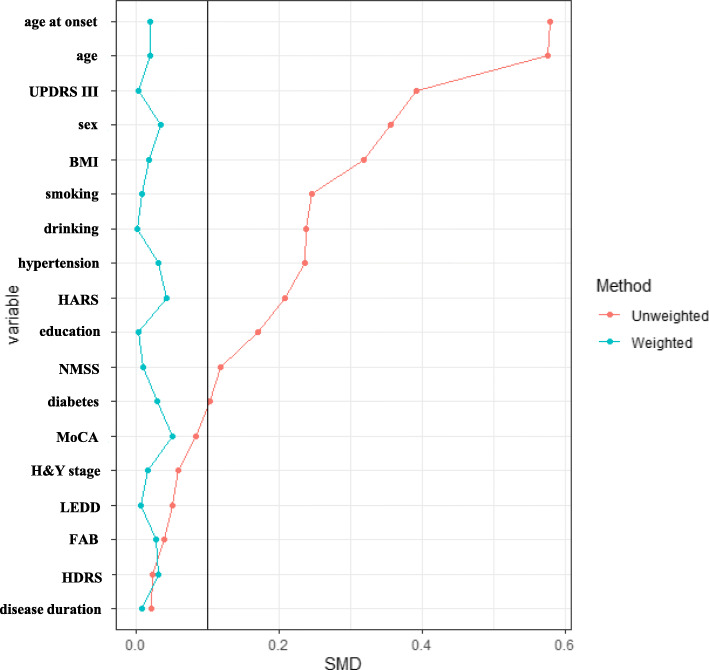
SMD in baseline covariates between patients with and without FT before and after IPTW. SMD of baseline confounding covariates that were included for estimating the propensity scores before and after weighting. All the values of SMD were < 10% after IPTW weighting, which indicated there was a between-group balance after weighting. UPDRS III: Unified Parkinson’s disease Rating Scale part III. H&Y stage: Hoehn and Yahr stage. BMI: Body Mass Index. FAB: Frontal Assessment Battery. MoCA: Montreal Cognitive Assessment. HDRS: Hamilton Depression Rating Scale. HARS: Hamilton Anxiety Rating Scale. NMSS: Non-Motor Symptoms Scale. LEDD: levodopa equivalent daily dosage. SMD: Standardized Mean Differences. FT: facial tremor. IPTW: inverse probability of treatment weighting

IPTW was formed by those with complete data on clinical outcomes. A total of 18 covariates at baseline including sex, age, age of onset, disease duration, drinking, smoking, hypertension, DM, BMI, education, LEDD, UPDRS III score, H&Y stage, MoCA score, FAB score, HDRS score, HARS score, and NMSS score were included for estimating the propensity score (Fig. 3). Compared to pre-weighting, the SMD values of each variable at baseline were reduced after-weighting. All the SMD values were < 10%, suggesting there was a between-group balance on baseline characteristics after weighting (Fig. 3 and Supplementary Table 2).

The univariate Cox model indicated that baseline FT and FT as the initial symptom in the unweighted PD population were associated with survival (*P* = 0.006 and *P* < 0.001, respectively), but this association disappeared after using the weighted method by IPTW (*P* = 0.166 and *P* = 0.342, respectively) (Table 2). Baseline FT and FT as the initial symptom were also not associated with time to UPDRS-III 11-point increase, time to convert to H&Y stage 3, time to dyskinesia, and time to MoCA 3-point decrease both before and after matching (*P* > 0.05) (Table 2). In the sensitivity analysis, the multivariate Cox regression model suggested that baseline FT and FT as the initial symptom were not associated with the five clinical outcomes after adjusting confounding factors (*P* > 0.05, both in model 1 and model 2) (Table 3).

**Table 2:** Univariate Cox model for exploring the association between FT and clinical outcomes in PD before and after weighting

	Unweighted sample		Weighted sample	
	HR (95%CI)	*P*-value	HR (95%CI)	*P*-value
Presence of FT at baseline				
Time to death	0.41 (0.22–0.80)	0.006*	0.62 (0.31–1.22)	0.166
Time to UPDRS-III 11-point increase	0.77 (0.43–1.30)	0.339	0.98 (0.54–1.76)	0.934
Time to conversion to H&Y stage ≥3	0.98 (0.50–1.78)	0.938	0.66 (0.33–1.30)	0.224
Time to dyskinesia	0.41 (0.14–0.95)	0.061	0.44 (0.17–1.17)	0.101
Time to MoCA 3-point decrease	0.93 (0.51–1.61)	0.793	0.67 (0.36–1.23)	0.198
FT as initial symptom				
Time to death	2.82 (1.55–5.11)	< 0.001*	1.75 (0.55–5.54)	0.342
Time to UPDRS-III 11-point increase	0.97 (0.60–1.55)	0.889	0.74 (0.24–2.31)	0.606
Time to conversion to H&Y stage ≥3	1.24 (0.69–2.12)	0.467	0.34 (0.08–1.56)	0.167
Time to dyskinesia	0.51 (0.21–1.27)	0.149	0.81 (0.18–3.70)	0.790
Time to MoCA 3-point decrease	1.04 (0.63–1.72)	0.885	0.45 (0.13–1.63)	0.226

*FT* Facial tremor, *PD* Parkinson’s disease, *UPDRS* Unified PD Rating Scale, *MoCA* Montreal Cognitive Assessment

* Significant difference

**Table 3:** Multivariate Cox regression model for exploring the association between FT and clinical outcomes in patients with PD

	Model 1		Model 2	
	HR (95%CI)	*P*-value	HR (95%CI)	*P*-value
Presence of FT at baseline				
Time to death	1.65 (0.87–3.12)	0.122	1.50 (0.78–2.90)	0.230
Time to UPDRS-III 11-point increase	1.23 (0.75–2.01)	0.421	1.21 (0.73–2.00)	0.460
Time to conversion to H&Y stage ≥3	0.95 (0.52–1.74)	0.876	0.93 (0.51–1.72)	0.822
Time to dyskinesia	0.64 (0.25–1.61)	0.340	0.57 (0.23–1.45)	0.241
Time to MoCA 3-point decrease	0.97 (0.57–1.64)	0.903	0.97 (0.57–1.65)	0.907
FT as initial symptom				
Time to death	2.46 (0.90–6.76)	0.080	2.56 (0.91–7.23)	0.075
Time to UPDRS-III 11-point increase	0.91 (0.33–2.52)	0.861	0.86 (0.31–2.42)	0.777
Time to conversion to H&Y stage ≥3	0.47 (0.11–1.96)	0.300	0.49 (0.12–2.06)	0.332
Time to dyskinesia	1.07 (0.25–4.47)	0.931	1.09 (0.26–4.63)	0.906
Time to MoCA 3-point decrease	0.54 (0.17–1.74)	0.301	0.50 (1.16–1.63)	0.253

*FT* Facial tremor, *PD* Parkinson’s disease, *UPDRS* Unified PD Rating Scale, *MoCA* Montreal Cognitive Assessment

Model 1, adjusted for sex, age, and age at onset

Model 2, adjusted for sex, age, age at onset, BMI, LEDD, UPDRS III score, MoCA score, and NMSS score

## Discussion

We explored the prevalence and clinical correlates of FT in a large cohort of patients with PD. In a sample of patients who were assessed at “off” medication, FT was present in 18.1% of cases and independently associated with females, older age, and longer disease duration in addition to the core motor symptoms. Furthermore, we also assessed the prognostic value of FT on disease progression of PD and found that both the presence of FT and FT as the initial symptom were not associated with survival, dyskinesia, as well as motor and cognitive decline.

The prevalence of FT reported by previous studies [5, 25] was lower than our findings. In a previous study that included patients with disease duration < 2 years, the authors revealed that only 1.5% (4/263) patients had FT [25]. Another study included 50 patients with longer disease duration (mean 9.0 ± 6.6 years) and found 14% of patients had FT [5]. The strength of the current study is that we recruited a large sample population with all H&Y stages. Moreover, our study disclosed that the prevalence of FT was increased with advanced disease duration and H&Y stage, which can explain the complexity of symptomatology of the disease in the advanced stage and is likely to increase the difficulty to manage this symptom over time. Our findings also indirectly confirm that the evidence proposed that the tremor in PD may spread to other sites with the progression of the disease [2].

In the current study, we found that FT was associated with older age and longer disease duration. Although the likelihood of developing FT increases over time in patients with PD, the age and duration of the disease itself cannot be totally responsible, because not all patients with PD will eventually develop tremors. Furthermore, it is interesting that we found that female patients showed more susceptibility to experience with FT, which should be further pathologically verified since men are reported at a higher risk for PD and present with a faster deterioration of motor and non-motor functions [26].

In looking for the clinical correlates of FT with other motor symptoms, we found that both rest and kinetic tremors on limbs were positively correlated with FT. Such association suggests tremors occurred in PD no matter in limbs or facial areas may share some common pathophysiological mechanisms. It is noteworthy that different body parts may have similar tremor frequencies in PD, but are generally not the same and are not phase-locked [27]. This suggests that each body part has a separate tremor generator. The ability to stay separation may be due to the somatotopic segregation of basal ganglia loops [27]. Furthermore, it must be emphasized that tremor in PD may have multifaceted phenomenology and, probably, pathophysiology [27].

The association between FT and speech disorders indicates that the neural network among these symptoms may connect. One explanation for such a phenomenon is that subthalamic stimulation can cause a significant deterioration of speech or improve loudness of speech while relieving tremor [28]. In addition, the negative association between rigidity and FT is probably due to the fact that patients with rest tremor usually presented with slighter rigidity compared to those who do not exhibit rest tremor [25]. The neurophysiological similarities between tremors and the cogwheel phenomenon usually associated with rigidity can explain such association [29]. Moreover, the greater improvement of tremor and rigidity by deep brain stimulation [30] is another possible explanation. However, lack of relationship between FT and bradykinesia, another cardinal motor sign of PD that is strongly responsive to dopaminergic drugs, and relates to the dopaminergic cell loss in the substantia nigra [31], supports a contribution of non-dopaminergic mechanisms involving in FT. Although the response to levodopa of FT was not assessed in the current study, lack of association between FT and LEDD supports the above hypothesis.

In the current study, we found that both baseline FT and FT as the initial symptom were not associated with motor and non-motor progression, suggesting that FT is not a prognostic indicator for monitoring the progression of PD. Based on the ratio of tremor and non-tremor score, patients with PD were usually classified as TD and posture instability gait disorder (PIGD) subtypes. It is reported that patients with the TD subtype have a more benign course compared to those with the non-TD subtype [9]. Based on our finding that FT does not contribute to the disease progression, it is better not to be considered FT as a contributor in the TD subtype when calculating the ratio of tremor and PIGD to classify the PD subtypes.

Actually, a recent study [11] verified the severity of PIGD could be more appropriate as a clinical biomarker for the disease progression and the authors also did not support the use of the TD subtype as a prognostic trait in PD. This is because several longitudinal studies found that patients may switch their initial motor subtype from TD to PIGD, a transition that seems unidirectional [31, 32]. Since patients with the PIGD subtype show faster progression than those with the TD subtype, and subtype classification of patients is based on a ratio score of tremor/non-tremor symptoms, the switch from the TD subtype to the PIGD subtype is basically driven by increased severity of PIGD. However, as we stated earlier, each body part has a separate tremor generator, so our study suggests that a more complicated pathophysiology may involve in the development of FT compared to rest tremor at hands. Therefore, the prognostic value of each type of tremor should not be based on the ratio method. Instead, the effect of tremor and PIGD severity should be individually evaluated.

Some limitations should be considered. First, we focused on a group of patients who were assessed at “off” medication and therefore selection bias cannot be ruled out. Second, the relatively short observation of FT on clinical outcomes is not enough to conclude the influence of FT on long-term outcomes such as death. Third, the assessment of FT was based on UPDRS, a scale that does not distinguish between lips and chin tremor, resulting in these variables could not be used for stratification.

## Conclusions

FT is not rare in patients with PD, which can be affected by sex, age as well as disease duration and motor severity. Baseline FT and FT as the initial symptom are not associated with the disease progression of PD. The predictive value of FT on long-term progronis of PD may need to clarify by future longer follow-up studies.

## Supplementary Information

**Additional file 1: Supplementary Table 1.** Diagnosis of multicollinearity for each candidate variate. **Supplementary Table 2.** SMD of baseline covariates before and after IPTW between groups

## Data Availability

All data generated or analysed during this study are available by reasonable requiry. (Please contact: Huifang Shang, email: hfshang2002@126.com).
