# Supplementary material for: Facial tremor in patients with Parkinson’s disease: prevalence, determinants and impacts on disease progression
Source: BMC Neurol. 2021 Feb 23;21:86. doi: 10.1186/s12883-021-02105-y (PMC7901083; doi:10.1186/s12883-021-02105-y)
Supplement: Supplementary file 1 — Additional file 1: Supplementary Table 1. Diagnosis of multicollinearity for each candidate variate. Supplementary Table 2. SMD of baseline covariates before and after IPTW between groups [file 12883_2021_2105_MOESM1_ESM.docx]

**Supplementary Table 1** Diagnosis of multicollinearity for each candidate variate

|  | Tolerance | VIF |
| --- | --- | --- |
| Education | 0.741 | 1.350 |
| Sex, male | 0.891 | 1.122 |
| Age | 0.833 | 1.200 |
| Disease duration | 0.679 | 1.472 |
| Speech | 0.566 | 1.765 |
| Facial expression | 0.543 | 1.840 |
| Rest tremor on hands | 0.759 | 1.317 |
| Action or postural tremor | 0.807 | 1.239 |
| Rigidity | 0.425 | 2.351 |
| Bradykinesia | 0.302 | 3.308 |
| Axial symptoms | 0.394 | 2.539 |
| FAB | 0.501 | 1.996 |
| MoCA | 0.432 | 2.317 |
| NMSS | 0.701 | 1.426 |

FAB: frontal assessment battery. MoCA: Montreal Cognitive Assessment. NMSS: Non-Motor Symptoms Scale. * Significant difference.

**Supplementary Table 2 SMD of baseline covariates before and after IPTW between groups**

|  | FT as initial symptom (y/n) | |
| --- | --- | --- |
|  | unweighted | weighted |
| Age | 0.735 | 0.012 |
| Age at onset | 0.732 | 0.009 |
| Disease duration | 0.102 | 0.034 |
| Sex | 0.671 | 0.004 |
| BMI | 0.420 | 0.012 |
| Education | 0.164 | 0.009 |
| LEDD | 0.246 | 0.006 |
| Smoking | 0.668 | <0.001 |
| Drinking | 0.428 | 0.003 |
| Hypertension | 0.008 | 0.018 |
| Diabetes | 0.124 | <0.001 |
| UPDRS III | 0.222 | 0.010 |
| H&Y stage | 0.238 | 0.006 |
| FAB | 0.314 | 0.054 |
| MoCA | 0.428 | 0.006 |
| HDRS | 0.500 | 0.015 |
| HARS | 0.399 | 0.017 |
| NMSS | 0.506 | 0.008 |

SMD of baseline confounding covariates that were included for estimating the propensity scores before and after weighting. All the values of SMD were < 10% after IPTW weighting, which indicated there was a between-group balance after weighting.

SMD: Standardized Mean Differences. FT: facial tremor. IPTW: inverse probability of treatment weighting. BMI: Body Mass Index. LEDD: levodopa equivalent daily dosage. UPDRS III: Unified Parkinson’s disease Rating Scale part III. H&Y stage: Hoehn and Yahr stage. FAB: Frontal Assessment Battery. MoCA: Montreal Cognitive Assessment. HDRS: Hamilton Depression Rating Scale. HARS: Hamilton Anxiety Rating Scale. NMSS: Non-Motor Symptoms Scale.
